# The Foreign Journals: Midwifery

**Published:** 1839-04

**Authors:** 


### MIDWIFERY.

*Case of Pregnancy of the Fallopian Tube.* By Dr. BAMBERGER, of Berlin.

[We have already recorded several cases of this kind, (See particularly Vol. III. pp. 243-522,) but the interest of the subject is far from being exhausted.]

Philippine N., æt. 34, had spent ten years of a former marriage childless, and had already passed from three to four years of a second marriage without having a child. On the evening of the 4th of March, whilst playing at cards with a few friends, she suddenly left the room, and was found a few minutes afterwards sitting upon the sofa in another room, complaining of faintness and nausea, which she ascribed to a pain in the abdomen, which she had begun to feel about half an hour before. The tongue was coated, the pulse scarcely perceptible, and the face deadly pale. The pain in the abdomen was not very violent, and was accompanied by a bearing down in the rectum. The patient was disposed to ascribe her complaints to the delay of the menses, which should have appeared six or seven days before; the bowels were constipated, but the other functions were in a normal state. Vomiting ensued in about a quarter of an hour, and removed almost all the uneasiness, although the face still continued very pale, and the pulse was small, wiry, and quick. At eleven she went to bed, assuring her friends that she felt perfectly well. She could not, however, fall asleep, and the vomiting returned several times through the night. At four in the morning she complained of pain in the abdomen, especially in the gastric region. and of a feeling of tightness reaching from the stomach to the neck, which rendered every movement painful. The abdomen felt somewhat tense, and was painful on pressure, but the gastric region was more sensible than the parts below the umbilicus. The pulse was quick and wiry, and the cold perspiration stood on the forehead and limbs. As there had been no motion of the bowels, castor oil was prescribed, and an injection administered; cupping-glasses were at the same time applied to the abdomen. In consequence of these measures the tenseness of the abdomen was somewhat diminished; and another injection succeeded in producing motion of the bowels, and a quantity of hard scybala were passed. The condition of the patient, nevertheless, did not improve; small quantities of ice were now given to check the vomiting, and leeches were applied to the abdomen. She slept some hours during the night, but the vomiting still continued. At twelve she became very low, and one fainting fit succeeded upon another, till she expired about half-past seven in the evening.

On examining the body, a large quantity of blood was found in the cavity of the abdomen, and the left fallopian tube was burst about its middle by an ovum about the size of a pigeon's egg. The fœtus was not found in the ovum, and it had probably escaped into the cavity of the abdomen.

Dr. Bamberger was at first inclined to ascribe the symptoms to obstruction of the bowels; but he was afterwards obliged to assume the presence of internal hemorrhage, although he could not determine its cause. He appears to have suspected that extra-uterine pregnancy might have been the cause of the phenomena; but not to have attached much value to this supposition, as the pain was less acute than he would have suspected in pregnancy of the tube; and the characteristic cry, as described by Heim, (See Br. and For. Med. Rev., Vol. IV. p. 493,) was wanting. *Casper's Wochenshrift*, No. 39. 1838.

---

*Two Cases of Episioraphy, to elucidate a Modification of the Operation.* By Dr. FRICKE, of Hamburg.

CASE I. This woman had already been successfully operated upon about fifteen months before, on account of a considerable prolapsus of the uterus. About two months before her admission, on the 8th March, 1837, she had been delivered of a full-grown child; but the accoucheur who had been called in to assist her had judged it necessary to divide the adhesions, consequent on the former operation, in order to facilitate labour. The natural result was a second prolapsus to an extent which prevented all exertion.

The edges of the labia were made raw and approximated to each other, and held in position by a number of sutures. The modification in the operation consisted in leaving a free space (in this case accidentally produced), of about half an inch in extent, at the anal commissure, by which the menstrual and other secretions could escape, without accumulating and causing inconvenience. Six weeks after the operation the woman left the hospital completely relieved.

CASE II. J. A., aged 41, admitted 6th July, 1837, had suffered during the last six years from prolapsus of the uterus, which followed on difficult parturition, during which the forceps were applied. The protruded uterus and vagina were about the size of a child's head; the mucous membrane of the vagina was smooth, dry, and leathery, excoriated in many parts, and covered with hard scabs. Several days' rest, and preliminary treatment were necessary before the uterus could be replaced; and seven days afterwards, the menses, which had not appeared for years, began to flow.

On the 22d August, the operation was performed as described in the preceding case, except, that here a space, sufficient to admit the finger, was purposely left free at the anal commissure. The union was complete in fourteen days, except a small part at the posterior edge of the wound, which speedily healed on touching it occasionally with caustic. The prolapsus was completely retained.

The advantages resulting from this modification of the operation, are:

1. It is less painful and is more quickly performed.
2. Local antiphlogistic remedies are more easily applied.
3. Injections into the vagina escape readily through the orifice.
4. Mucus and the menstrual fluid are not retained.

*Zeitschrift für die gesammte Medicin.* Band viii. Heft 2. *Hamburg.*

